# Baby-Led Weaning: What Role Does It Play in Obesity Risk during the First Years? A Systematic Review

**DOI:** 10.3390/nu13031009

**Published:** 2021-03-21

**Authors:** Nazareth Martinón-Torres, Nathalie Carreira, Rosaura Picáns-Leis, Alexandra Pérez-Ferreirós, Anton Kalén, Rosaura Leis

**Affiliations:** 1 Unit of Pediatric Gastroenterology, Hepatology and Nutrition, Pediatric Service, University Clinical Hospital of Santiago (CHUS), 15706 Santiago de Compostela, Spain; nazareth.martinon.torres@sergas.es (N.M.-T.); nathalie.carreira.sande@sergas.es (N.C.); 2 Pediatric Nutrition Research Group, Institute of Sanitary Research of Santiago de Compostela (IDIS), CHUS-USC, 15706 Santiago de Compostela, Spain; 3 Department of Pediatrics, University Clinical Hospital of Santiago (CHUS), 15706 Santiago de Compostela, Spain; rosaurapicansleis@gmail.com; 4 Unit of Investigation in Human Nutrition, Growth and Development of Galicia (GALINUT), University of Santiago de Compostela (USC), 15701 Santiago de Compostela, Spain; alexandra15pf@gmail.com (A.P.-F.); anton.kalen@gmail.com (A.K.); 5 CIBEROBN, (Physiopathology of Obesity and Nutrition) Institute of Health Carlos III (ISCIII), 28029 Madrid, Spain

**Keywords:** overweight, weight gain, infants, complementary feeding, body mass index, childhood, infant nutrition, feeding behavior

## Abstract

Childhood is a window of opportunity for the prevention of the obesity pandemic. Since “the first 1000 days of life” is a period in which healthy eating habits must be acquired, it should be the target for preventive strategies. Baby-led weaning (BLW) is an emergent way of weaning that could influence children’s health. The nutrition committees of the main pediatric societies affirm there is not enough evidence to support which is the best method of weaning. The aim was to determinate the influence of BLW on the infant’s weight gain compared to the traditional spoon-feeding, and to assess if it could decrease the risk of obesity in children. A systematic review was conducted, following the PRISMA method. Pubmed, Web of Science, Embase, and Cochrane Library were searched. Out of 747 articles, eight studies (2875 total infants) were included (two randomized control trials, 6 observational studies). Results were indecisive, while some studies seem to demonstrate lower weight gain in infants that apply BLW, others show inconclusive results. The risk of bias in all included studies was moderate or high. In conclusion, more clinical trials and prospective studies should be done prior to providing a general recommendation about the best method of weaning to reduce the risk of obesity.

## 1. Introduction

Prevalence of overweight and obesity in children has increased dramatically in the last four decades, from 4% to 18%, becoming one of the pandemics of the 21st century [1]. Overweight children have an increased risk of being overweight as adults, leading to a higher risk for suffering from respiratory, metabolic and cardiovascular diseases than children with normal weight [1,2]. There are multiple risk factors for the development of overweight in infancy, both genetics and environmental [2]. Some of these environmental factors, such as dietary habits and physical activity, seem to act from the very early stages of life, and thus it is a current challenge for the scientific community to identify them early and be able to act preventively [3].

“The first 1000 days of life” refers to the period between the moment of conception and the child’s second birthday and is a dynamic period of extreme vulnerability for the growth and development of the child [3]. Therefore, this period appears to be the best target for preventive strategies against later medical conditions as overweight and obesity [3,4,5,6]. Nutritional intervention in the infant and the establishment of healthy eating patterns from the beginning of life are some of those possible strategies. In this sense, feeding in “the first 1000 days” of life seems to play an important role on children’s development and on later adult health outcomes. There is quite significant evidence on the role of breastfeeding in terms of these effects [1,3,7,8]. Several benefits of breastfeeding on children’s health have been found, e.g., providing some level of protection against childhood overweight and obesity, although the underlying mechanisms remain unknown. However, there is less evidence on the effect of the rest of the infant’s diet on childhood overweight and obesity [9,10,11].

The World Health Organization (WHO) recommends to exclusively breastfeed infants for the first six months of life, as human milk provides all the energy and nutrients they need for a proper growth [7]. This recommendation was changed at the beginning of the century based on systematic reviews on the estimated energy intake of healthy term-born infants that were breastfeeding and growing well [12]. From around six months of age, other foods besides milk must be introduced to the infant’s diet for nutritional and developmental needs [7,13,14,15]. The weaning, or starting of complementary feeding, must be understood as a period rather than a specific moment. For the specific timing of introducing complementary feeding, the infant’s nutritional needs, degree of psychomotor, gastrointestinal and renal development must be taken into account. In any case, the introduction should not be made before 17 weeks of age, as recommended in a position paper by the European Society for Paediatric Gastroenterology, Hepatology and Nutrition (ESPGHAN) [13]. The nutritional committees of the main Pediatric Societies affirm that there is not enough evidence to determine which is the best method of weaning: either the traditional introduction of grinded or mashed food using a spoon (called traditional spoon-feeding or standard weaning) or the increasingly popular baby-led weaning (BLW), in which a variety of whole foods are presented to the infant, who self-select them and self-feed [7,13,14,15].

Since the first references to the BLW by Rapley [16], some studies have suggested and observed the possible benefits of this weaning method on infants’ health [17,18]. It has been proposed that BLW could favor the infant’s appetite control and lead to higher levels of satiety-responsiveness, thus contributing to protect against later overweight, although with controversial results [17,18]. Despite BLW gaining popularity, together with its potential benefits on health outcomes, there is very little high-quality evidence on this method of weaning reaching several different conclusions. Concerns about the risk of not ingesting enough nutrients (especially iron and zinc) for the proper infant weight gain and growth by this method [19,20], as well as the risk of choking, have also been pointed out by other authors [21].

The importance of finding ways to reduce childhood overweight and obesity has already been pointed out. Strategies applied in the very early stages of life can have a great impact on the future health of children. Intervening in how to introduce complementary feeding can be one of them. BLW, a weaning method different from the traditional spoon-feeding, has been proposed to have many benefits including protection against later overweight [17]. To our knowledge, there is not any systematic review specifically assessing the effect of BLW on the infants’ weight in comparison with the traditional spoon-feeding approach. Therefore, the aim of this review was to determinate the influence of BLW on the infant’s weight gain compared to the traditional spoon-feeding, and to assess if it could decrease the risk of obesity in children. The analysis of the current evidence could contribute to better knowledge of which is the best weaning approach to prevent overweightness and obesity, one of the most challenging public health issues today.

## 2. Materials and Methods

This review was designed following PRISMA (Preferred Reporting Items for Systematic Reviews and Meta-Analyses) guidelines and was registered in the International Prospective Register of Systematic Reviews (PROSPERO) [22]. The review question we tried to answer was: Does the baby-led weaning approach decrease the risk of obesity in children? It was formulated in accordance with the PICOS (population, intervention, comparison, outcomes and study type) criteria (Table 1) [23].

### 2.1. Literature Search

Searches were made in the Pubmed, Web of Science, Embase and Cochrane Library in March 2021. Due to the lack of specific Medical Subject Headings (MeSH) terms for “baby-led weaning”, searches in PUBMED were conducted using the following search terms: (“weaning” (MeSH) OR “baby-led approach” OR BLW OR “baby-led weaning”) AND (“pediatric obesity” (MeSH) OR “Overweight” (MeSH) OR “body mass index” OR “body weight” OR “body composition”) AND (child OR infant OR children OR childhood) NOT (“Ventilator Weaning” OR “tube-feeding” OR “Premature Birth” OR animal OR rat OR mice). In the other databases, the same search terms were used, changing MeSH terms to regular terms.

### 2.2. Inclusion and Exclusion Criteria

Articles considered for inclusion were any controlled trial and observational study that compared weight or body mass index (BMI) in BLW and spoon-fed infants of any age, published in English or Spanish language from 2000 to March 2021. We excluded other types of publications like review articles, guidelines, letters, commentaries, or books, although some of them were included as a part of the discussion. Other exclusion criteria were: do not address the infant weight or body mass index as an outcome or unavailability of the full text.

### 2.3. Intervention

Studies considered for inclusion were those involving infants that followed BLW as a method of weaning; regardless of definition of BLW, age at the start of complementary feeding and duration of the intervention. In the randomized controlled trials (RCTs), the BLW group was considered the intervention group.

### 2.4. Outcome Measures

Studies considered for inclusion were those that provided data about infants’ weight or BMI, regardless of whether it was self-reported or measured by the research team.

### 2.5. Study Selection

From the 917 articles obtained from the database searches and other sources, two independent examiners (N.M.-T. and N.C.) selected studies for inclusion. Throughout all this selection process, discrepancies were arbitrated by two other researchers (R.L. and R.P.-L.). Finally, 8 studies were included in this review [24,25,26,27,28,29,30,31]. 

### 2.6. Data Extraction

From each article included, the following data were extracted by the two independent examiners: reference, publication year and location, type of study, size of the sample and dates of recruitment, age of the infants, outcome measures, intervention (when applicable), method of weaning and definition of BLW, and main results and conclusions in relation to the aim of our review.

### 2.7. Assessment of Risk of Bias

Following the methodology of the Cochrane handbook [32,33], two authors (A.K. and A.P.-F.) independently assessed the risk of bias in the included articles, with any unresolved disagreements discussed with a third author (R.L.) who made the final decision if consensus could not be reached. For RCTs, the RoB 2 tool was used to assess the risk of bias in five domains: randomization process, assignment to intervention, missing outcome data, measurement of the outcome, and selection of the reported results [34]. For the observational studies, the ROBINS-I tool was used to assess the risk of bias in seven domains: confounding, selection of participants, classification of interventions, deviations from intended interventions, missing data, bias in measurement of outcomes, and selection of reported results [35].

## 3. Results

**Figure 1:**
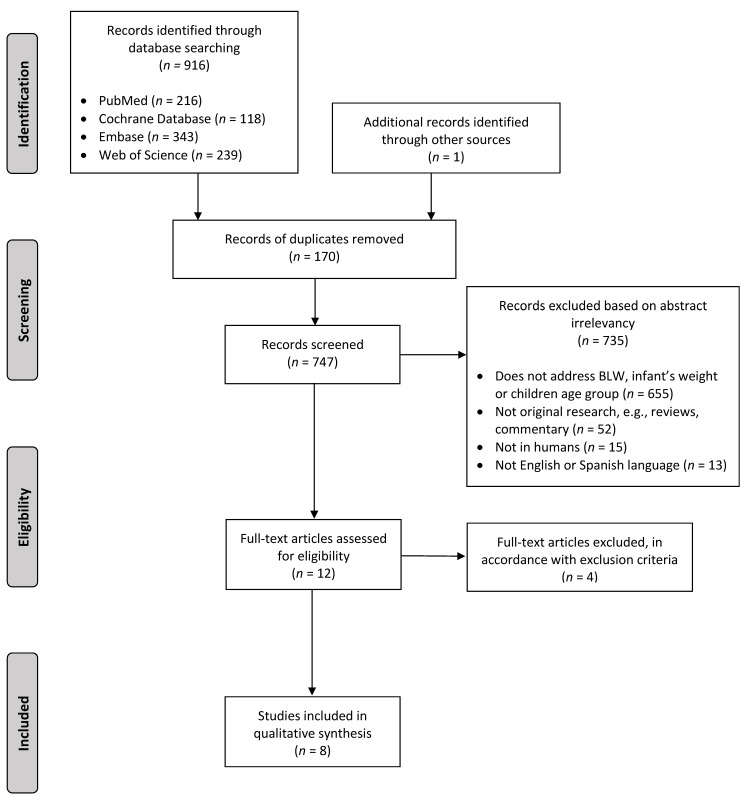
PRISMA Flow Diagram of the literature search and selection, according to the inclusion and exclusion criteria.

Figure 1 shows the process followed for the selection of the studies. A total of 916 articles were identified through database searches, and one additional was identified through screening of reference lists. After removing duplicates, title and abstract of 747 articles were screened. Of these, 735 were excluded: 655 did not address BLW, infant’s weight or children age group, 52 were not original studies, 15 used non-human sample, and 13 were not written in English or Spanish. Of the 12 full-text articles assessed for eligibility, four were excluded, giving a final of eight included articles in the review [24,25,26,27,28,29,30,31].

### 3.1. Study Characteristics

Table 2 shows the main characteristics of the eight studies included: two RCTs [26,28] and six observational studies (four cross sectional [24,27,30,31], one longitudinal [29], and one with both cross-sectional and longitudinal analysis [25]). They were ordered according to the year of publication, starting with the most recent. All studies were published between 2011 and 2020. The recruitment or data collection ran from June 2006 to February 2018. Infants were recruited through posters calling in community halls, family centers and baby groups [25,31], posts on relevant internet sites or online parenting forums [24,27,29,30], laboratory databases [30], a Well Child Clinic [26] and a maternity hospital [28]. Four of the studies were located in the United Kingdom [25,29,31], two in New Zealand [27,28], and the other two in Turkey [24,26]. The age of the study populations ranged from birth to 78 months.

In total, the eight studies included 2875 infants of whom 486 participated in randomized controlled trials [26,28] and 2120 provided data through on-line questionnaires answered by their parents [24,27,29,30,31]. The sample of one of the observational studies [29] was constituted of a subgroup of 298 children that acceded to a follow up (Phase 2, longitudinal analysis) from a previous study on 604 mothers that was also included in this review (Phase 1, cross-sectional analysis) [31].

### 3.2. Method of Weaning

Of the total 2875 infants included, 1430 followed BLW approach (339 of them, a partial BLW); all the rest followed a traditional spoon-feeding approach. Definition of BLW varied between the included articles. In two of the observational studies, infants were classified as baby-led weaners if they reported using both spoon feeding and purees 10% of the time or less [29,31]. In another of the observational studies, participants were asked to select frequency of spoon-feeding using a five-point scale and were grouped as “predominantly self-fed” if self-fed always and often, or “predominantly spoon-fed” if self-fed sometimes, rarely or never [25]. Fu et al. [27] classified the infants in three categories: traditional spoon-feeding, partial BLW or full BLW, depending on being mostly spoon-fed, half self-fed and half spoon-fed, or mostly self-fed. In the study by Townsend and Pitchford [30], parents self-reported the weaning style used (BLW or traditional spoon-feeding) and responded to some control questions on weaning methods to check the accuracy of their answer. In the most recent observational study [24], parents self-identified as following BLW, BLW plus traditional weaning, or only traditional weaning, although there are not definitions for these terms in the text. In both the RCTs included in this review [26,28], the intervention group received training and support on a modified form of BLW that encourages offering iron-rich and energy-dense foods and avoiding foods with high risk of chocking [36].

In relation to possible confounders, all articles [24,25,26,27,28,29,30,31] mention the breastfeeding rate, but only Jones et al. [25] consider this variable for their analysis, and Dogan et al. [26] uses breastfeeding as inclusion criteria.

### 3.3. Weight and Body Mass Index

All the studies included measures of infants’ weight, in five it was self-reported by their parents [24,27,29,30,31] (in one of them as recorded from a health professional in the infant’s “Well Child” book [27]) and in three it was measured by the research team according to a systematic protocol at least once [25,26,28]. In five of the studies, nutritional indexes were calculated (BMI in four of them [24,25,28,30], weight-for-length in one [26]) and the percentiles and z-score for the values calculated based on the WHO child growth reference data [37] in all but two [24,30]. BMI percentile ranks in the study by Townsend et al. were calculated using the Centers for Disease Control and Prevention (CDC) Child and Teen BMI calculator and the National Health Service (NHS) Choices BMI Calculator (which uses UK90 reference data for children older than four years and WHO Growth Standards data for children younger than four years) [30]. BMI, weight and height Standard Deviation Score (SDS) in the study by Kahraman et al. were calculated using the work of Neyzi et al., for the values of Turkish children [24]. 

In the RCT done by Dogan et al. [26], traditional spoon-fed infants had gained more weight than BLW group at 12 months of life (11.1 ± 0.5 kg vs. 10.4 ± 0.9 kg). By this time, no overweight was seen in BLW vs. 17% in traditional spoon-feeding. Underweight was only demonstrated in 2% of BLW infants. By contrast, Taylor et al. [28] does not find significant differences between groups, despite high prevalence of overweight in BLW group.

The findings in the observational studies varied. Two of them [29,30] found lower BMI in BLW children compared to spoon-fed infants. In the Brown et al. study [29], overweight prevalence was higher in traditional spoon-feeding (19.2% vs. 8.1%) as in the one by Kahraman et al. [24] (14.7% vs. 5.1%). One of them [24] linked weight gain in traditional spoon-feeding group with formula feeding, while the other two [27,31] did not reach any significant conclusions.

### 3.4. Risk-of-Bias Assessment

Of the two included randomized control trials, the Dogan et al. [26] study had overall some concerns regarding risk of bias. This came from the fact that the pre-registration of the trial could not be located. Meanwhile, the Taylor et al. [28] study was assessed to show a high risk of bias, as reasons for drop-out and missing data was not clearly stated. Of the six observational studies, three show a moderate, and three a serious risk of bias. Of the six observational studies, three showed a high overall risk of bias, which is due to confounding [27,29,30]. Further, all six studies showed a moderate risk of bias in selection of reported results, as none used any type of pre-registration. Full results of the risk-of-bias assessment can be seen in Supplementary Figures S1–S4.

## 4. Discussion

This systematic review of RCTs and observational studies assessed the effect of using a BLW compared to traditional spoon-feeding approach on the risk of obesity in infants. Eight articles with a total of 2875 infants were included, two RCTs [26,28] and six observational studies [24,25,27,29,30,31]. There was considerable variation in the results between studies, and no reliable recommendations could, therefore, be established. The results of the observational studies can be influenced by different factors and must, therefore, be interpreted with caution. The risk of bias is moderate-to-high in all the studies. 

We only identified two RCTs exploring the effect of a BLW approach on anthropometric measures in infants compared to those following a traditional spoon-feeding approach [26,28]. The intervention group in both trials received specific training support on a modified form of BLW called Baby-Led Introduction to Solids (BLISS), that encourages offering iron-rich and energy-dense foods, and avoiding foods with high risk of chocking [36].

In the most recent of these RCTs, Dogan et al. [26] recruited 280 breast-fed infants between 5 and 6 months of age and found that infants in the traditional spoon-feeding group were significantly heavier compared to those in the BLW group at 12 months of age. Using a stepwise linear regression, they found type of weaning to influence the weight at 12 months independently of other covariates, such as birthweight and duration of exclusive breastfeeding. These differences in the infants’ weight between the BLW and traditional spoon-feeding groups were not seen by Taylor et al. in a previous RCT [28]. Taylor et al. recruited 206 women in late pregnancy that were randomized to follow a baby-led or a spoon-feeding approach [28]. They measured the infants’ weight and length for a longer time, up to 24 months, and did not find statistically significant differences in the BMI z-score at neither 12 or 24 months of age between the BLW and the control groups. Unlike the results of Dogan’s RCT, the prevalence of overweight in the BLW group was higher than in the control group, both at 12 and 24 months.

Previous observational studies had shown very different results to these seen by Taylor et al. [28], and more similar to those seen by Dogan et al. [26]. An example of this is the study by Townsend and Pitchford [30], in which they found a significantly lower BMI in the BLW group compared to the traditional spoon-feeding group in a cohort of 155 children aged 20 to 78 months. Another example is the longitudinal study by Brown et al. in which they observed that the infants in the traditional spoon-feeding group were significantly heavier than those in the BLW group at 18–24 months [29]. The same trend was seen in a recent study by Kahraman [24], with a higher prevalence of overweightness in the traditional weaning infants. Taylor et al. [28] suggested that the most likely explanation for these different findings regarding the infants’ weight among the BLW and traditional spoon-feeding groups could be related to the study design (observational studies vs. RCT) and to the use of the modified form of BLW (BLISS), that may have attenuated the risk of growth faltering [36]. However, these factors could not explain, by themselves, the discrepancies in the results between the two RCTs [26,28]. Besides the method of weaning, there are probably other factors that can influence the infants weight gain. Prolonged breastfeeding and delay in the introduction of complementary foods have been associated to a lower risk of overweight in infants in many studies [1,7,38,39]. Considering this, in a recent observational study examining the impact of the method of weaning upon infant growth during the first year of life, Jones et al. [25] took into account the type of milk feeding, and observed that spoon-feeding was associated with increased infant weight only among formula-fed. Dogan et al. [26] also considered the possible influence of this factor, since breastfeeding was an inclusion criteria for their RCT, but still found that 17% of the infants in the traditional spoon-feeding group were overweight at 12 months. Therefore, there should probably be more factors besides the method of weaning and the type of milk feeding, such as the energy intake, the energy self-regulation or demographic variables, that should also be considered when assessing the infants’ weight gain. 

There are some differences in the design of the two RCTs that need to be considered when comparing them. Unlike the RCT by Dogan et al., breastfeeding was not an inclusion criterion in Taylor et al. [28]. The median duration of exclusive breastfeeding in the last one was shorter in both BLW and traditional spoon-feeding groups, although baby-led infants breast-fed for more time than controls, as previously reported in other studies [40,41]. It has already been pointed out the probable influence of many factors in the infants’ weight gain. Homogeneous rates of breastfeeding among the BLW and the traditional spoon-feeding groups in further RCTs could contribute to reduce the possible effect of these factors.

Another difference between the RCTs was the length of the follow-up. Infants were followed until 12 months of age by Dogan et al. [26] and up to 24 months by Taylor et al. [28]. In the last, they did not find significant differences between the BLW and control groups for BMI z-score neither at 12 or 24 months [28]. These results suggest the maintenance of the same effect of the method of weaning on BMI z-score for, at least, the first 2 years of life, although there was a loss of 19.4% of infants at 24 months. Some studies emphasize the strength of the influence on weight gain not only in the early infancy but also in the childhood and adolescence (especially during the period of adiposity rebound, at around 5 to 7 years) [42]. In this sense, Townsend and Pitchford is the only study that include older children, with a wide range of ages, up to 78 months [30]. However, when interpreting their results, we should be aware of the limitations of an observational study design and the possible inaccuracy of the parents self-reported weight of most of the infants included. Thus, it would be of interest to design RCTs that evaluate the weight trajectory among children with different methods of weaning not only in the first months of life but longer in the childhood, in order to see if the effect is maintained over time.

Many studies point the special importance of a rapid weight gain during infancy as a predictor of adiposity in later life [43,44]. Of the studies included in this review, only Jones et al. [25] calculated the weight gain velocity (change in weight-for-age z-score from birth to age at the time of measurement) and did not find significant differences between the two feeding groups. More studies measuring this index are necessary to be able to assess if the method of weaning has influence on their weight gain velocity.

Prevalence of overweight was higher in the traditional spoon-feeding than in the BLW group, with values up to 19.2%, in five of the studies included [24,25,26,29,30], while no cases of overweight were found in the RCT by Dogan et al. [26]. That’s why these authors suggest that BLW could contribute to protect infants against obesity. Regarding the prevalence of underweight, it was found to be higher in the BLW group, with an average of 5% in half of the studies included [25,29,30] compared to the traditional spoon-feeding group, with no cases in half of the articles [25,26,30]. 

It is important to highlight that regardless the disparity of results in these studies, children’s weight and nutritional indexes, when calculated, showed normal values for their gender and age in most cases (according to the WHO child growth references) [37]. In the RCT by Dogan et al. [26], 98% of the infants in the BLW group and 83% of those in the traditional spoon-feeding group had a normal weight-for-length z-score at 12 months. Similar percentages of normal BMI z-scores were observed in both the BLW and the traditional spoon-feeding groups in the study by Jones et al. [25], in the one by Kaharman [24] and a bit lower but still high, according to the weight-for-age z-score, in Brown et al. (86.5% in the BLW group and 78.3% in the traditional spoon-feeding group) [29].

In any case, caution must be taken when drawing conclusions from this review since it includes eight studies, of which only two were RCTs. Further, all the included studies showed at least some concerns regarding the risk of bias. The main issue was that only one of the eight studies was pre-registered and accessible. Other reasons for the increased risk of bias were the failure to control for relevant confounders in observational studies and failing to give detailed information regarding dropout and missing data in RCTs. Caution should also be taken given the small sample-size in most of the studies, the different data source (measurements made by search team or self-reported) and because the nutritional indexes considered are not the same in all de studies included, and therefore not quite comparable.

Given the big interest that exist around BLW, it is remarkable that we only found eight articles studying the effect of BLW on the risk of infants’ obesity. Further, in the nearly three years since the last systematic review on BLW, only three new studies were published on the subject. There is, therefore, a big need for more studies, and especially RCTs, on the matter. Future studies should preferably use pre-registration, include weight gain velocity, use longer follow-up periods and control for important confounders, as birthweight or the duration of breastfeeding.

There are multiple factors that must be taken into account when analyzing obesity risk. The type, time, and approach of complementary feeding could play an important role in the obesity prevention at later age. Breast-feeding duration and other confounders must also be taken into account. It is important to note that the lack of evidence about the benefits of BLW causes doubts in the handling of feeding by parents.

The results of this systematic review about the effect of BLW on infant weight gain and overweight prevalence are inconclusive. More clinical trials and longer prospective longitudinal studies should be done, using confounder controls (e.g., birthweight and breastfeeding) and including several body composition variables and weight gain velocity, to show which feeding technique is the best to reduce obesity risk in childhood.

## Figures and Tables

**Table 1:** PICOS (Population, Intervention, Comparison, Outcome and Study type) criteria for the inclusion of studies.

Parameter	Inclusion Criteria
Population	Infants and children (no limitation for age)
Intervention	Baby-led weaning approach
Comparison	Standard or spoon-fed weaning approach
Outcome	Weight, body mass index and prevalence of overweight
Study type	Controlled trial and observational studies

**Table 2:** Main characteristics of the studies included in the review.

Reference	Location	Type of Study	N (Number of Subjects) Recruitment (Dates)	Age of Infants (Months)	Outcome Measures	Definition of BLW	Intervention	Results and Conclusions (Regarding Weight or Body Mass Index)
Kahraman et al. (2020) [24]	Turkey	Observational (cross-sectional)	485 mothers (137 BLW, 246 partial BLW, 102 TSF) October 2017–February 2018.	6–24	Self-reported infant weight and length. Calculation of WAZ, LAZ and BMIZ (Turkish children reference data).	(No definition in the text)	None	Less overweight in BLW (BMIZ > 2SD: 5.1% BLW vs. 14.7% TSF; *p* = 0.000).
Jones et al. (2019) [25]	United Kingdom	Observational (comparative cross-sectional and longitudinal)	Cross-sectional study: 269 infants (109 BLW, 160 TSF). Longitudinal study: 101 infants (30 BLW, 71 TSF). February 2016–November 2017.	3–12	Infant weight and length at one time point (cross sectional data set) and ≥16 weeks later (longitudinal data set). Calculation of WAZ, LAZ, BMIZ and WAZV (WHO reference data).	Predominantly self-fed (self-fed always and often).	None	No significant differences in WAZ among BLW regardless of the type of breastfeeding (WAZ, mean (SD): =−0.07 (1.35) BLW and formula vs. 0.06 (1.00) BLW and any breastfeeding; *p* = 0.84) TSF and fully formula fed (both simultaneously) significantly heavier than those who had received any breasfeeding (WAZ, mean (SD): 0.38 (0.81) TSF and formula vs. 0.17 (0.98) TSF and any breastfeeding; *p* = 0.04) Lower increases in WAZ and BMIZ in BLW, but not statistically significant (WAZ change: +0.13 BLW vs. +0.29 TSF, *p* = 0.86; BMIZ change: +0.17 BLW vs. +0.42 TSF, *p* = 0.07)
Dogan et al. (2018) [26]	Turkey	RCT	280 breast-fed infants (142 BLW, 138 Control (TSF). January 2014–April 2016.	5/6–12	Infant weight, length and head circumference (at 6 and 12 months). Calculation of weight for length percentiles (at 12 months; WHO reference data).	BLISS	Control (TSF): standard well childcare. BLW group: 4 additional group training meetings and 6 home visits for support and education in BLISS until 11 months of age.	Control (TSF) significantly heavier at 12 months (11.1 ± 0.5 kg Control (TSF) vs. 10.4 ± 0.9 kg BLW; *p* < 0.001). No overweight in BLW at 12 months (BMIZ > 2SD: 17% in Control (TSF) vs. 0% in BLW). No underweight in Control (TSF) at 12 months (BMIZ ≤ 2SD: 0% in Control (TSF) vs. 2% in BLW).
Fu et al. (2018) [27]	New Zaeland	Observational (cross-sectional)	876 parents (155 BLW, 93 partial BLW, 628 TSF) June 2017.	6–36	Infant weight at 6–7 months as recorded by a health professional. Calculation of WAZ.	Mostly self-fed or self-fed	None	No differences in WAZ between full BLW and TSF at 6–8 months. (WAZ, mean (SD): −0.3 (0.9) BLW vs. −0.4 (1.3) TSF; *p* = 0.874).
Taylor et al. (2017) [28]	New Zealand	RCT	206 women in late pregnancy (105 BLW, 101 Control (TSF)). 166 infants at 24 months (88 BLW, 78 Control (TSF)). December 2012–March 2014.	0–24	Infant weight (at 6, 7, 8, 9, 12, and 24 months) and length (6, 12 and 24 months). Calculation of BMI and BMIZ (at 12 and 24 months; WHO reference data).	BLISS	Control (TSF): government-funded routine midwifery and well-childcare. BLW group: 8 additional contacts (6 face-to-face, 2 telephone) for lactation and BLISS support from birth to 9 months of age.	No significant differences in BMIZ between BLW and Control (TSF) at 12 months (adjusted difference, 0.23; 95% CI, −0.06 to 0.52) and at 24 months (adjusted difference, 0.15; 95% CI, −0.12 to 0.45). Higher risk (no statistically significant) of overweight in BLW at 12 months (RR 2.5; 95% CI, 0.9 to 6.9) and at 24 months (RR 1.8; 95% CI, 0.6 to 5.7).
Brown and Lee (2015) [29]	United Kingdom	Observational (longitudinal)	298 children from a previous study [31] (163 BLW, 135 TSF).	18–24	Self-reported infant weight and length at the time of the recruitment. Calculation of WAZ (WHO reference data).	≤10% spoon-feeding and purees.	None	TSF significantly heavier at 18–24 months. (Weight, kg (SD): 12.86 kg (3.73) in TSF vs. 11.79 kg (3.53) in BLW; *p* = 0.005). Less overweight in BLW at 18–24 months (8.1% BLW vs. 19.2% TSF). More underweight in BLW at 18–24 months (5.4% BLW vs. 2.5% TSF).
Townsend and Pitchford (2012) [30]	United Kingdom	Observational (case-control)	155 parents (92 BLW, 63 TSF). June 2006–January 2009.	20–78	Self-reported infant weight and length. Calculation of BMI, BMIZ (WHO reference data) and BMI percentile rank (CDC, NHS).	Self-reported as BLW (and checked by specific questions about their weaning practices).	None	Lower BMI in BLW (CDC percentile rank: 48.46 BLW vs. 61.44 TSF; *p* = 0.009). Less obesity in BLW (BMIZ > 2: 1.6% BLW vs. 12.7% TSF). More underweight in BLW (BMIZ ≤ 2: 4.8% BLW vs. 0% TSF).
Brown and Lee (2011) [31]	United Kingdom	Observational (cross-sectional)	604 mothers (351 BLW, 253 TSF).	6–12	Self-reported estimates of the infant weight (at 6 months and at the time of recruitment). Self-reported perception of infants’ growth (at 6 months).	≤10% spoon-feeding and purees.	None	No significant differences for estimated weight at 6 months or estimated current weight (statistics not available).

Abbreviations: RCT, randomized controlled trial; BLW, baby-led weaning; BLISS, Baby-Led Introduction to Solids; TSF, traditional spoon-feeding; WAZ, weight-for-age z-score; LAZ, length-for-age z-score; BMI, body mass index; BMIZ, body mass index z-score; WAZV, weight gain velocity for age-z-score; WHO, World Health Organization; NHS, National Health Service; CDC, Centers for Disease Control and Prevention.

## Data Availability

Not applicable.

## Supplementary Materials

The following are available online at https://www.mdpi.com/2072-6643/13/3/1009/s1, Figure S1: Result of risk of bias assessment for each randomized control trial using the RoB 2 tool, Figure S2: Result of risk of bias assessment for each observational study using the ROBINS-I tool, Figure S3: Summary of risk of bias assessment for randomized control trials using the RoB 2 tool, Figure S4: Summary of risk of bias assessment for observational study using the ROBINS-I tool.
