# Supplementary material for: Baby-Led Weaning: What Role Does It Play in Obesity Risk during the First Years? A Systematic Review
Source: Nutrients. 2021 Mar 21;13(3):1009. doi: 10.3390/nu13031009 (PMC8003981; doi:10.3390/nu13031009)
Supplement: Supplementary file 1 [file nutrients-13-01009-s001.pdf]

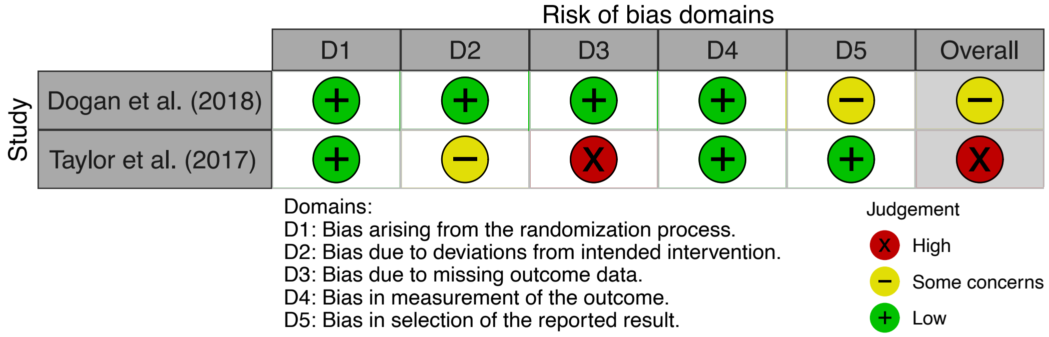

Figure S1. Result of risk of bias assessment for each randomized control trial using the RoB 2 tool.

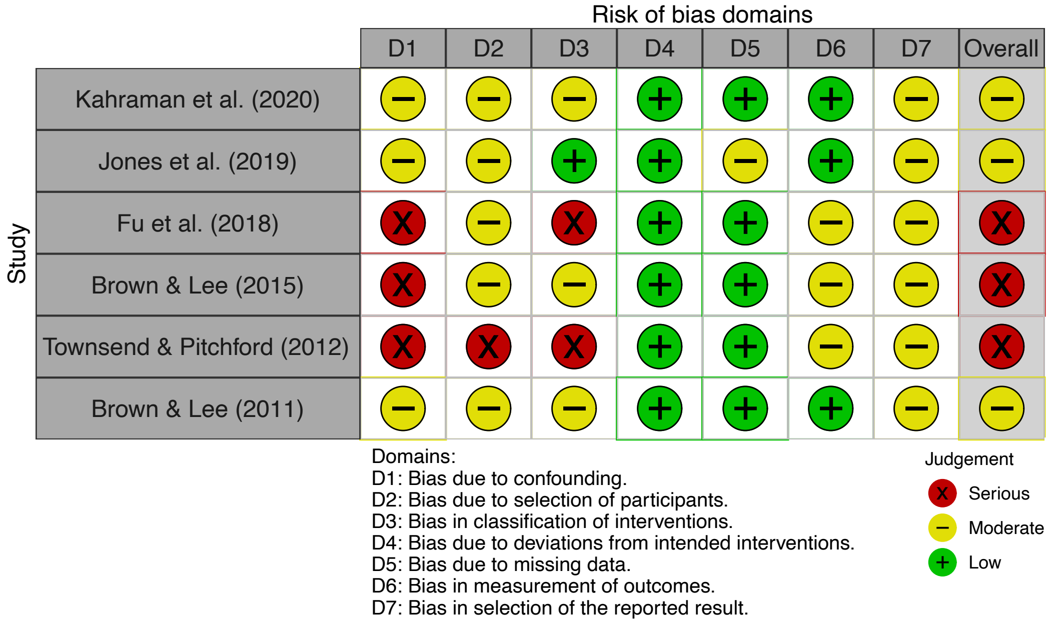

Figure S2. Result of risk of bias assessment for each observational study using the ROBINS-I tool.

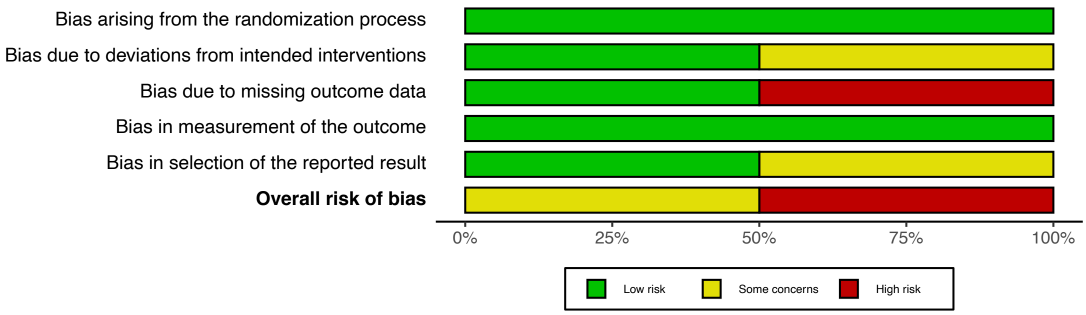

Figure S3. Summary of risk of bias assessment for randomized control trials using the RoB 2 tool.

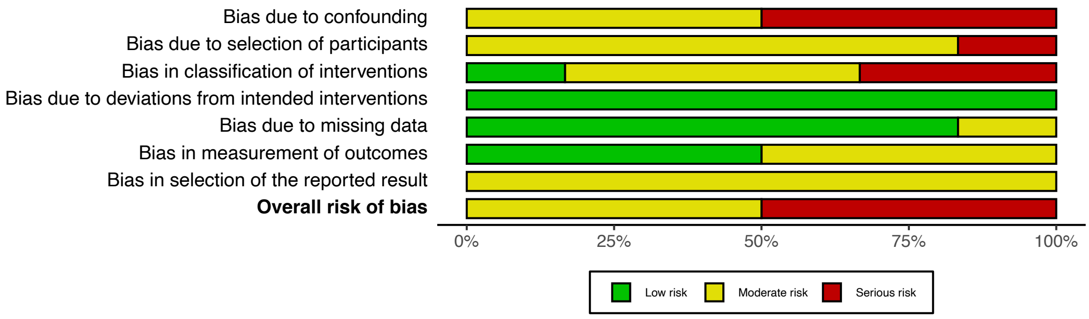

Figure S4. Summary of risk of bias assessment for observational study using the ROBINS-I tool
